# Nutrient and acetate amendment leads to acetoclastic methane production and microbial community change in a non‐producing Australian coal well

**DOI:** 10.1111/1751-7915.12853

**Published:** 2017-09-19

**Authors:** Michiel H. in 't Zandt, Sabrina Beckmann, Ruud Rijkers, Mike S.M. Jetten, Mike Manefield, Cornelia U. Welte

**Affiliations:** ^1^ Department of Microbiology Institute for Water and Wetland Research Radboud University Heyendaalseweg 135 6525 AJ Nijmegen The Netherlands; ^2^ Netherlands Earth Systems Science Center Utrecht University Heidelberglaan 2 3584 CS Utrecht The Netherlands; ^3^ School of Chemical Engineering School of Civil and Environmental Engineering University of New South Wales High Street 2052 Sydney NSW Australia; ^4^ Soehngen Institute of Anaerobic Microbiology Radboud University Heyendaalseweg 135 6525 AJ Nijmegen The Netherlands

## Abstract

Coal mining is responsible for 11% of total anthropogenic methane emission thereby contributing considerably to climate change. Attempts to harvest coalbed methane for energy production are challenged by relatively low methane concentrations. In this study, we investigated whether nutrient and acetate amendment of a non‐producing sub‐bituminous coal well could transform the system to a methane source. We tracked cell counts, methane production, acetate concentration and geochemical parameters for 25 months in one amended and one unamended coal well in Australia. Additionally, the microbial community was analysed with 16S rRNA gene amplicon sequencing at 17 and 25 months after amendment and complemented by metagenome sequencing at 25 months. We found that cell numbers increased rapidly from 3.0 × 10^4^ cells ml^−1^ to 9.9 × 10^7^ in the first 7 months after amendment. However, acetate depletion with concomitant methane production started only after 12–19 months. The microbial community was dominated by complex organic compound degraders (*Anaerolineaceae*,* Rhodocyclaceae* and *Geobacter* spp.), acetoclastic methanogens (*Methanothrix* spp.) and fungi (Agaricomycetes). Even though the microbial community had the functional potential to convert coal to methane, we observed no indication that coal was actually converted within the time frame of the study. Our results suggest that even though nutrient and acetate amendment stimulated relevant microbial species, it is not a sustainable way to transform non‐producing coal wells into bioenergy factories.

## Introduction

Coal is the most important fossil fuel on the planet, comprising 70% of the total fossil fuel stock (Iram *et al*., 2017). Coal mining has a big environmental impact and is estimated to result in 24–42 Tg of methane emissions per year. Methane in coalbeds is produced biologically or thermogenically and escapes from both active and abandoned coal mines. This amounts to 11% of total yearly anthropogenic methane emissions (Saunois *et al*., 2016) and thus has a considerable climate impact due to the high global warming potential of methane (34 for 100 years, (Myhre *et al*., 2013)). Coalbed methane can be used as a more environmentally sustainable alternative to direct coal burning (Fakoussa and Hofrichter, 1999; Al‐Jubori *et al*., 2009; Kinnon *et al*., 2010) as direct coal burning leads to pollution in the form of heavy metals and sulfur compounds (Querol *et al*., 1995). At the same time, the exploitation of coalbed methane could effectively reduce methane leaking into the atmosphere. In many cases, coalbed methane harvesting is not profitable due to unfavourable system properties, like highest rank coal species or unfeasible methane production rates as well as barriers to a selling market (Moore, 2012). As a result, many active methane‐producing coal systems are neglected and thus remain sources of methane emission.

Biogenic coalbed methane production requires a consortium of anaerobes that convert coal compounds to substrates for methanogenic archaea (Jones *et al*., 2010). Recently, Mayumi and co‐workers showed that coal‐derived methoxylated compounds can be directly used for methanogenesis by *Methermicoccus* species (Mayumi *et al*., 2016). Coal biosolubilization, the rate limiting step, is carried out by fungi expressing peroxidases, laccases, hydrolytic esterases and also abiotically by alkaline metabolites and naturally occurring chelators (Fakoussa and Hofrichter, 1999; Strąpoć *et al*., 2008; Papendick *et al*., 2011). The microbial degradation of intermediates to volatile fatty acids (VFAs) and the subsequent production of acetate and H_2_/CO_2_ fuels the methanogens (Robbins *et al*., 2016).

In Australia, roughly 5000 coal seam gas wells were operated in 2014 (Day *et al*., 2014) which accounted for 18% of the total gas production in 2014–2015 and almost half of the total East Coast gas production (Ball *et al*., 2016). Several studies focused on enhancing methane production by applying nutrient, trace element and biotic amendment to low‐emitting coal seams (Jones *et al*., 2010; Penner *et al*., 2010; Ünal *et al*., 2012). In Australia, coalbed exploitation for biogas production has gained more attention in the last decade and several studies focused on characterizing and enriching microorganisms from coal (Faiz and Hendry, 2006; Midgley *et al*., 2010; Papendick *et al*., 2011; Robbins *et al*., 2016). However, detailed data on the effects of nutrient amendments on microbial communities in these systems are scarce but highly needed to successfully optimize coalbed methane production.

In this study, we analysed methane production, cell counts, physicochemical properties of the well water and acetate degradation in a sub‐bituminous coal well over the course of 25 months. We compared this data to the microbial community composition at 17 months when acetate consumption and methane production were observed and at 25 months, the final time point, using 16S rRNA gene amplicon sequencing. For the 25 month sample, the functional potential of the microorganisms was investigated using a metagenome data set. This data set was also interrogated to study the fungal, archaeal and bacterial communities using ribosomal rRNA gene based phylogeny. By combining this set of complementary experiments, we could reconstruct the complex microbial food web and assess the suitability of nutrient and acetate amendment to transform non‐producing coal seams into gas production sites.

## Results and discussion

The two experimental coal wells are located at the Lithgow State Coal Mine in the Western Coal Fields of New South Wales, Australia. The coal bearing layers are located 80 m below ground level. About 2 m^2^ of coal is continuously exposed to the groundwater that has a volume of 450 L, a pH of 7.9, an oxidation reduction potential of −232 mV (chemical potential relative to the standard hydrogen electrode (SHE)) and a temperature of 16°C. Groundwater flows were not assessed here but they likely cause exchange and mixing of the water body. After 25 months of operation, the pH was 8.2 and the redox potential −197 mV. The headspace gas volume is circa 1000 L. The well was nutrient amended by adding a final concentration of 1.8 mM ammonium and 1.9 mM phosphate in addition to 20 mM acetate in two 10 mM additions at 0 and 4 months. Nutrient and acetate concentrations were pre‐assigned according to preliminary *in vitro* culture assays performed and modified according to Jones *et al*. (2010). Nutrient and acetate solutions were slowly released by drop tubing to 450 L coal formation water in contact with the coal seam with a feeding rate of about 0.5 L min^−1^ for a 2 L stock solution.

### Amendment stimulated microbial growth and methane production

**Figure 1:**
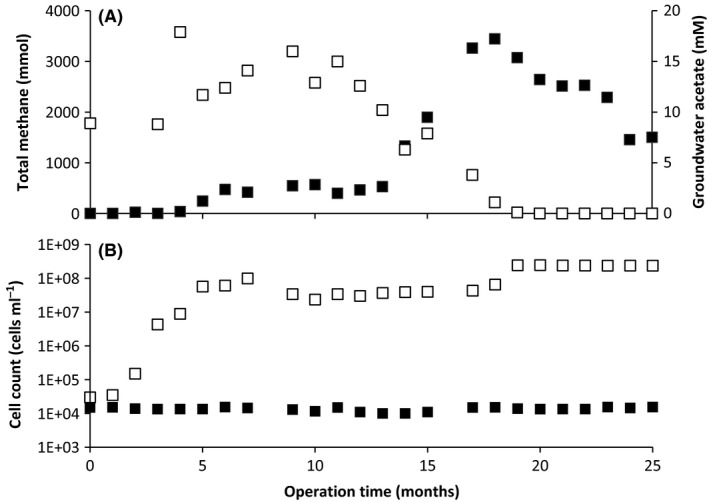
Monthly methane and acetate concentration measurements in the amended coal well (A) and total cell count data for both the nutrient/acetate amended and the control coal well (B). No methane production was observed in the control coal well. To the amended coal well, 10 mM acetate was added at 0 and 4 months respectively. A: open squares (□), acetate concentration in mM; filled squares (■), total methane in mmol; B: open squares (□), total cell count in cells ml^−1^ for the nutrient/acetate amended well; filled squares (■), total cell count in cells ml^−1^ for the control well.

The amended well showed considerable methane production after 12 months, whereas in the unamended well, no methane production was observed over the course of the whole experiment (25 months, Fig. 1A). These observations are in line with previous studies on microbial coal conversion that showed that nutrient amendment stimulated methane production from non‐producing coal systems (Laborda *et al*., 1997; Jones *et al*., 2010; Penner *et al*., 2010). Interestingly, methane emission was only observed after a prolonged lag phase of 12 months in conjunction with acetate degradation and stopped when acetate was depleted after about 18 months. This indicates that the microbial community at the start of the experiment was not yet able to or not abundant enough to convert acetate to methane. When we analysed the cell numbers in the two coal wells, we found that there was considerable growth in the amended well in the beginning of the incubation (3.0 × 10^4^ cells ml^−1^ at 0 months; 9.9 × 10^7^ cells ml^−1^ at 7 months, Fig. 1B) with a stabilization of the cell density after 7 months (2.4 × 10^8^ cells ml^−1^ at 25 months) and absence of growth in the control well (1.5 × 10^4^ cells ml^−1^ at 0 months; 1.6 × 10^4^ cells ml^−1^ at 25 months, Fig. 1B). The increase in cell numbers was not correlated with a decrease in acetate concentration or concomitant methane production so it was presumably mainly due to the addition of nutrients that were also limiting in the unamended coal well. After about 11 months, acetate consumption and methane production were first observed, indicating a shift in microbial community functioning. From this, it can be concluded that acetate and nutrient amendment clearly stimulated microbial growth and enabled the microbial community to eventually convert acetate to methane. As methane production stopped after acetate depletion, these data also suggest that the methane‐producing microbial community was not able to thrive without external addition of acetate (Fig. 1A) even though the microbial biomass had increased by several orders of magnitude (Fig. 1B). Stoichiometric analysis indicated that about 25% of the acetate was converted to methane, suggesting presence of syntrophic acetate oxidation, which has been previously observed in organic carbon‐rich petroleum reservoirs (Mayumi *et al*., 2011). Sulfate‐dependent acetate oxidation was unlikely to occur due to the rapid depletion of sulfate from 1 mM at the start of the experiment (Table S1) to below the detection limit after 3 months (< 0.02 mM). The decrease in methane after 19 months in the amended coal wells is probably due to escape of methane from the coal well rather than active degradation of methane. We analysed the sequencing data for aerobic or anaerobic methanotrophs but did not find any indication (16S rRNA gene and marker genes *pmoA*,* mcrA*) of aerobic or anaerobic methane oxidation.

**Figure 2:**
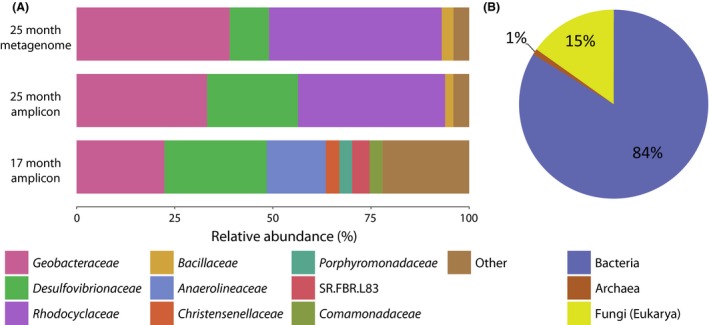
Phylogenetic analysis of the microbial community at 17 and 25 months based on 16S/18S rRNA genes. A. Bacterial taxonomy using 17 month and 25 month amplicon 16S rRNA gene data and 25 month metagenome bacterial 16S rRNA reads. B. Relative abundance of bacteria, archaea and fungi (eukarya) based on total metagenome 16S/18S rRNA gene mapped reads at 25 months.

To get insight into the functioning of the microbial food web and as indication as to why methane production stopped after 15 months, we sampled the coal well water at 17 and at 25 months to analyse the microbial communities and their functional potential. Phylogenetic analyses were performed on both 16S and 18S rRNA genes and fungal ITS (Fig. 2, Table S2). Functional analyses targeted genes encoding proteins involved in complex organic compound degradation, metal reduction, electron transfer reactions and volatile fatty acid metabolism (Table S2).

### The fungal community has the potential for coal biosolubilization

Fungi represented 15% of the combined 16S/18S rRNA gene reads detected in the metagenome data set (Fig. 2). The sequences were most related to species within the Agaricomycetes class that contains well‐characterized wood‐rotting fungi (Ohm *et al*., 2014). Additional identification based on fungal ITS sequences supported identification up to the Basidiomycota division, and most reads (89%) were closely related to *Basidiomycota* sp. MEL 2363319 (KP311433) (99% nt identity). In the metagenome, we found functional genes encoding lignin, manganese and versatile peroxidases that are involved in complex organic compound degradation. Even though these enzymes require oxygen as co‐substrate, Agaricomycetes have been previously detected in several anoxic environments such as marine ecosystems (reviewed by Manohar and Raghukumar, 2013), anoxic organic carbon‐rich mangrove soils (Arfi *et al*., 2011) and anoxic freshwater sediments (Zhang *et al*., 2015). This suggests that extremely low oxygen concentrations possibly penetrating into anoxic habitats can still support such enzyme activities. Agaricomycetes are the only class of fungi capable of substantial lignin degradation (Floudas *et al*., 2012), which supports their potential role in the breakdown of lignin‐derived structures in coal as has been showed for filamentous fungi (Hofrichter *et al*., 1997).

### Community shifts from *Anaerolinaceae* to *Rhodocyclaceae* as dominant potential complex organic compound degraders

At 17 months, the *Anaerolinaceae* were quite abundant (15% of 16S rRNA gene reads, Fig. 2) but almost completely disappeared at 25 months (< 0.2%). They were replaced by the *Rhodocyclaceae* comprising the genera *Azoarcus* and *Thauera* that were virtually absent at 17 months (< 0.4% of 16S rRNA gene reads) but became dominant community members at 25 months (23% of bacterial 16S rRNA gene reads in the amplicon survey; 44% relative abundance in the metagenome, Fig. 2). As there is no metagenome from the 17 month sample available and *Anaerolinaceae* were nearly absent at 25 months, a functional potential can only be inferred. Interestingly, their potential in coal degradation has not been suggested previously. However, *Anaerolineaceae* were dominant after long‐term enrichments with long‐chain n‐alkanes‐dependent methanogenic conditions and occur in combination with sulfate reducers and methanogens (*Methanoculleus* and *Methanothrix*) (Liang *et al*., 2015, 2016). In addition, enrichment from aquifer sediment with naphthalene (a polycyclic aromatic hydrocarbon) showed high relative abundance of Anaerolineaceae (5.7%–47.0%) and experimental evidence showed that polycyclic aromatic hydrocarbon (PAH) dioxygenases are present in *Rhodocyclaceae* and play a role in PAH metabolism (Singleton *et al*., 2012; Chemerys *et al*., 2014). The *Rhodocyclaceae* family members *Azoarcus* and *Thauera* consist of anaerobic aromatic compound degrading denitrifiers (Anders *et al*., 1995; Liu *et al*., 2006). As the absolute cell counts did not change considerably between the two sampling points of 17 and 25 months (Fig. 1B), it is interesting to note that the microbial community shifted towards a higher abundance of *Rhodocyclaceae*. This might indicate that the microbial community changed towards degrading complex organic compounds from coal biosolubilization. The involvement of both betaproteobacterial species in the degradation of aromatic compounds was supported by the presence of genes encoding benzylsuccinate synthase (BssABC) and 4‐hydroxybenzoyl‐CoA reductase (HcrAB) that previously have been shown to be involved in benzoate and toluene degradation respectively (Hermuth *et al*., Table 2002; Barragán *et al*., 2004) (S2). For *Azoarcus,* the functional analyses revealed the presence of benzoate‐coenzyme A ligase (BzdA) which supported its capacity for benzoate degradation (Barragán *et al*., 2004). The identification of *Thauera aromatica* was possible to species level and was supported by both metagenomic 16S rRNA and functional gene analyses. Its capacity for complex organic compound degradation including relatively inert aromatic compounds may be crucial in coal breakdown processes (Hermuth *et al*., 2002; Boll, 2011; Kuntze *et al*., 2011). Both *Azoarcus* and *Thauera* have been observed before in sub‐bituminous and bituminous methane‐producing coal seams (Li *et al*., 2008). Strikingly, we did not detect nitrate in the coal well at the start of the experiment (Table S1) so it is unclear what terminal electron acceptors could have been used by *Thauera* and *Azoarcus*.

### Organic compounds could be further degraded by *Geobacter metallireducens*

Both the 17 and 25 month amplicon sequencing data indicated high relative abundance of *Geobacteraceae* (22% and 33% of bacterial amplicon reads, respectively, Fig. 2). The 25 month metagenomic data supported the high relative abundance (32% of 16S rRNA reads, Fig. 2) and allowed the identification of a single *Geobacter* species present, namely *Geobacter metallireducens* (100% sequence identity). *G. metallireducens* inhabits freshwater sediments and couples metal reduction of mainly iron and manganese to the complete anaerobic oxidation of a wide variety of organic electron donors including acetate, butyrate, ethanol, butanol, propanol and aromatic hydrocarbons like toluene and phenol (Lovley and Phillips, 1988; Lovley *et al*., 2004; Aklujkar *et al*., 2009; Kuntze *et al*., 2011; Zhang *et al*., 2013). In *G. metallireducens,* carbon fluxes can be balanced via the acetate kinase/phosphotransacetylase (ACK/PTA) pathway that converts acetyl‐CoA to acetate with the generation of ATP, as shown for *Escherichia coli* (el‐Mansi and Holms, 1989). This mechanism is present in *G. metallireducens* and is hypothesized to be used when excess organic compounds are present (Lovley and Chapelle, 1995; Aklujkar *et al*., 2009; Speers and Reguera, 2012). Whether this pathway can provide acetate for methanogenesis in coal wells should be further investigated. Sequence analyses of metal reduction and complex organic compound degradation genes supported the potential role of *G. metallireducens* in these processes (all with 100% protein sequence identity, Table S2). Fe(III) reduction potential was analysed based on cytochromes involved in iron reduction and electron transfer mechanisms across the membrane. We found the diheme cytochrome *c* peroxidase MacA in the *G. metallireducens* draft genome (100% aa identity). In *G. sulfurreducens,* MacA is involved in the electron transfer pathway to Fe(III) (Butler *et al*., 2004; Seidel *et al*., 2012). We also detected the triheme cytochrome *c7* Gmet_2902 (homologous to *G. sulfurreducens* PpcA). Previous studies in both *Geobacter* species established that these cytochromes are essential for electron transfer reactions but do not directly reduce Fe(III) (Afkar and Fukumori, 1999; Lloyd *et al*., 2003). As a candidate for Fe(III) reduction we identified a gene encoding Gmetc6, a homologue of a putative *c*‐type outer membrane cytochrome (OmcF), that showed 100% protein sequence identity to the 74 aa conserved domain, indicating presence of an outer membrane electron transfer mechanism (Kim *et al*., 2005; Mehta *et al*., 2005). Genes encoding geopilin (PilA) and flagellar proteins for motility (FliCDS) were found and indicated the capacity for chemotaxis, direct substrate contact and importantly also electron transfer (Childers *et al*., 2002; Tremblay *et al*., 2012; Holmes *et al*., 2016). These factors can provide competitive advantages in the presence of difficult to dissolve Fe(III) and Mn(IV) minerals at high pH. Together with the capacity to oxidize aromatic hydrocarbons, this supports the potential role of *Geobacter* in metal reduction in the amended coal well.

### *Desulfovibrio* and *Bacillus* possess the capacity to couple organic compound oxidation to iron reduction

The amplicon data at 17 and 25 months revealed that *Desulfovibrio* accounted for 23% and 26% of the bacterial reads, respectively, whereas metagenome 16S rRNA reads indicated lower relatively lower abundance (10%) (Fig. 2). 16S rRNA and phylogenetic analysis of genes encoding sulfite reductase (DsvAB, the Dsr of *Desulfovibrio vulgaris*) revealed that *D. vulgaris* was the dominant organism. *Desulfovibrio vulgaris* belongs to a group of sulfate reducers that break down organic acids incompletely and produce acetate as end‐product (Heidelberg *et al*., 2004). However, sulfate was not detected after the first three months (< 0.02 mm) and FeS had accumulated in the sediment (data not shown). Upon sulfate depletion, *D. vulgaris* can switch to Fe(III) reduction, as well as to the reduction of Cr(VI) and U(VI) that occur as trace elements in sub‐bituminous coal (Querol *et al*., 1995; Heidelberg *et al*., 2004). However, whether this strategy can be used by *D. vulgaris* to compete for oxidized iron and to simultaneously produce acetate as substrate for acetoclastic methanogenesis should be further investigated.

*Baccillaceae* were nearly absent at 17 months (0.7%) and more abundant at 25 months (2% and 5% of amplicon and metagenome 16S rRNA reads, respectively, Fig. 2) with high 16S rRNA gene sequence identity to *Baccillus subterraneus*, a species that can use Fe(III), Mn(IV), nitrate, nitrite and fumarate as electron acceptors (Kanso *et al*., 2002; Strąpoć *et al*., 2008). A review by Nealson and Saffarini (1994) indicated that the iron and manganese reduction capacity of *Bacillus* species was more widespread (Nealson and Saffarini, 1994). Further work to assess coupling of metal reduction to organic compounds degradation and growth in *Bacillus* species is required.

### Acetoclastic *Methanothrix* (formerly: *Methanosaeta*) species are responsible for methanogenesis

Both at 17 and 25 months, *Methanosaetaceae* were the dominant archaeal family (≥ 99%). Based on the relative abundance data from the metagenome, they constitute 1% of the total microbial community (Fig. 2). Methane concentration measurements indicated highest methanogenic activity between 12 and 19 months after amendment. All members of the family *Methanosaetaceae* are strictly acetoclastic methanogens, indicating that hydrogenotrophic methanogenesis does not play a significant role in the amended coal well. BLASTX analysis of raw and *de novo* assembled reads that mapped to the methyl‐coenzyme M reductase protein subunit A (McrA) sequence database indicated high identity to both *Methanothrix concilii* GP‐6 (1 contig with 94% protein sequence identity) and uncultured methanogens predominantly found in lake sediments (Denonfoux *et al*., 2013; Youngblut *et al*., 2014; Lin *et al*., 2015; Pump *et al*., 2015). More specifically, the one assembled contig was very similar to the McrA sequence of an uncultured *Methanothrix* sp. from 90 m deep Lake Pavin sediment (95% protein sequence identity) (Denonfoux *et al*., 2013), in line with high similarity of the 16S rRNA gene sequence to uncultured *Methanothrix* species from Lake Pavin sediment and water samples (Lehours *et al*., 2005, 2007; Borrel *et al*., 2012). It is interesting to note that *Methanothrix* co‐occurred with *Geobacter*. A previous study by Jones *et al*. (2010) on bioaugmentation of sub‐bituminous coal showed degradation of single‐ring aromatics and long‐chain alkanes and subsequent methane production by a *Geobacter* spp. and *Methanothrix concilii* dominated culture. Experiments on microbial aggregates and co‐cultures showed that *Methanothrix‐Geobacter* clusters are electrically conductive which suggested that direct interspecies electron transfer (DIET) plays an important role (Summers *et al*., 2010; Morita *et al*., 2011; Rotaru *et al*., 2014; Holmes *et al*., 2017). These processes could also be relevant in the acetate and nutrient amended coal well.

### A community with low complexity has the potential for methanogenesis from coal but does not lead to successful continued methane production

**Figure 3:**
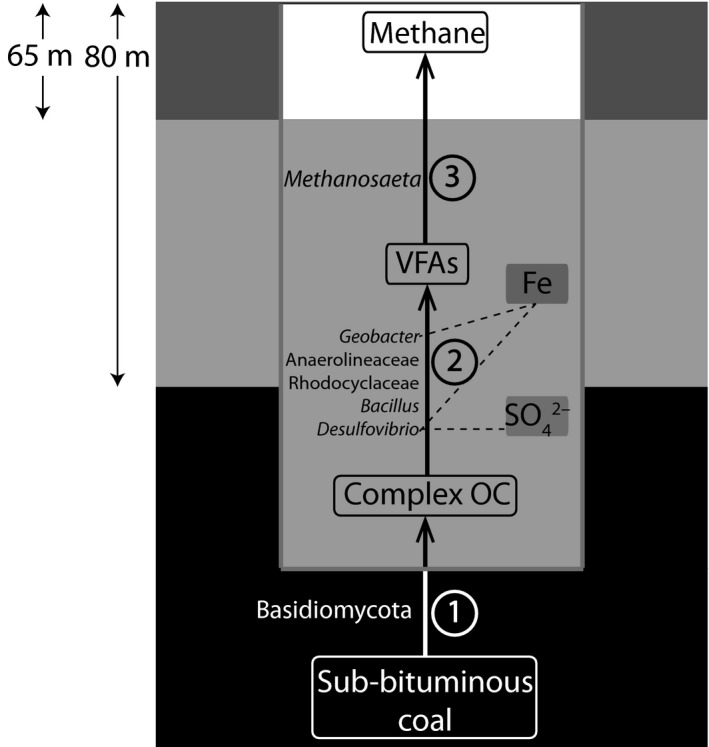
Proposed scheme illustrating the potential of the microbial community to convert sub‐bituminous coal to methane. The groundwater level is 65 m below ground level and the coal bearing layers are located 80 m below ground level. Fungi related to the Basidiomycota possess the capacity for coal biosolubilization. The bacterial community has the capacity for complex organic compounds degradation to volatile fatty acids (VFAs). Iron was detected as potential electron acceptor for *Desulfovibrio* and *Geobacter*, and sulfate could play a role only in the first three months. Acetoclastic *Methanothrix* spp. were the dominant methanogens in the coal well. Although the potential for bioconversion of the coal matrix was found, coalbed methanogenesis could not be observed.

Using geochemical, 16S rRNA gene amplicon and metagenome sequencing, we established that the microbial community in a nutrient and acetate amended Australian coal well had the potential to break down coal to methane (Fig. 3). The methane production data (Fig. 1A) indicated that methane was generated by acetate conversion rather than by coal degradation. It is puzzling that the microbial community primarily consisted of complex organic compound degraders, which would not have an apparent role in the conversion of acetate to methane. The second key observation is that nutrient amendment lead to an increase in microbial cell counts by four orders of magnitude, which did not result in direct methane production from coal. Degradation of dead microbial biomass could also play a role, but is unlikely to serve as a major organic compound source for methanogenesis. Both observations make it questionable whether nutrient and acetate amendment will help to sustainably generate coalbed methane from non‐producing coal wells, even if such amendments stimulate the microbial growth in general and help to bioaugment relevant microorganisms such as acetoclastic methanogens or complex organic compound degraders. In general, biological methane production from coal is a promising alternative to direct coal burning as it reduces inorganic and residual pollution in the form of heavy metals and sulfur compounds. From our microbial food web analysis coupled to the functional output of the nutrient and acetate amended coal well, it is apparent that it is not straightforward to link metabolic potentials to a system output. More *in situ* data on microbial activity, functioning and nutrient cycling are required to understand and overcome bottlenecks in the biomethanation process from coal.

## Experimental procedures

### Sample site

The two compared coal wells are located at the Lithgow State Coal Mine in the Western Coal Fields of New South Wales, Australia (Fig. S1). 30.6% of the raw sub‐bituminous coal in this study consisted of volatile hydrocarbons; over 50% consisted of fixed carbon (Mark Wainwright Analytical Centre, UNSW, Sydney, Australia). NH_4_Cl and Na_2_PO_4_ for the amendments were purchased from Ajax Finechem, Scoresby VIC, Australia and Sigma‐Aldrich, Castle Hill NSW, Australia respectively. For the nutrient and acetate additions, a 100 m × 0.5 mm (length × diameter) silicon tube was lowered to the base of the well (80 m below ground level) and concentrated nutrient solutions were gravity fed (siphoned) into the subsurface in syringe‐fed portions of 100 ml. During the feed, the tube was repeatedly manually raised and lowered from the bottom to the top of the water column (approximately 20 m) ensuring even distribution over the height of the water column. Lateral mixing was achieved by passive diffusion and disturbances imposed in sampling operations. By gravity flow the feeding rate was about 0.5 L min^−1^. Water sampling was performed using a well bladder pump (PVC 3/8 in discharge, ThermoFisher Scientific, Scoresby VIC, Australia) deployed with a stainless steel drop tube and a low refill ratio of 40:20 to avoid the removal of dissolved gases in the formation water. The drop tube was lowered to a depth of 75–78 m. The pump and connecting tubes were drained between the wells to avoid carriage of water between well sampling. Formation water for chemical and microbiological analyses was sampled monthly over the 25 month operation period. Samples were immediately processed as described below.

### Ionic composition of the well water

Analyses of cation and anion concentrations were carried out at the Mark Wainwright Centre (University of New South Wales, Sydney NSW, Australia). Briefly, cation (B, Ca, Fe, K, Mg, Mn, Na, P, S, Si) concentrations were analysed using inductive coupled plasma optical emission spectroscopy (ICP‐OES, Optima7300DV, Perkin‐Elmer, Waltham MA, USA) with a Segmented‐array Charged‐coupled Device (SCD, Perkin‐Elmer). The setting of the instrument was as follows: forward power 1200–1400 W, reflected power 20.0 W, nebulizer gas flow of 0.7 L min^−1^, plasma gas flow of 10.0–15.0 L min^−1^, aux gas flow of 0.3 L min^−1^. Anion (F, Cl, Br, NO_2_^−^, NO_3_^−^, PO_4_^3−^, SO_4_^2−^) concentrations were analysed using ion chromatography (IC, Dionex ICS1000, Dionex Corporation, Sunnyvale CA, USA) with conductivity detection using an Ion Pac AS14‐4 mm column (Dionex Corporation) and a mobile phase of 0.8 M Na_2_CO_3_/0.1 M NaHCO_3_. The instrument detection limits were 0.02 mg L^−1^ for B, Si and Mn, 0.2 mg L^−1^ for Fe and P, 0.5 mg L^−1^ for Ca, K, Mg, Na and S, 2 mg L^−1^ for F, NO_2_^−^, NO_3_^−^ and Br, and 5 mg L^−1^ for Cl and PO_4_^3−^.

### Total methane and acetate analyses

Total methane (headspace and dissolved) and acetate concentrations were monitored monthly over a period of 25 months. Gas samples were taken from the well‐head apertures and were transferred directly into 10 ml gastight serum vials using a gastight glass syringe. Dissolved methane in 100 ml sampled coal formation water was analysed according to Kampbell and Vandegrift (1998) with modifications. Briefly, formation water was sampled anoxically into 120 ml nitrogen degassed serum vials containing 1 ml of formic acid (Sigma‐Aldrich, Castle Hill NSW, Australia). Subsequently, samples were equilibrated to room temperature (20°C), and a 10 ml headspace was created in the vials by replacing 10 ml of liquid with 10 ml of nitrogen using a gastight glass syringe. Vials were inverted and equilibrated for 24 h. Methane from the headspace was analysed using a Shimadzu GC‐2010 gas chromatograph with flame ionization detection (GC‐FID) fitted with a GASPRO PLOT column (60 m × 0.32 mm; Agilent Technologies, Mulgrave VIC, Australia). The carrier gas was helium (3 ml min^−1^), and inlet temperature was 250°C. Oven temperature program: isothermal 100°C (1 min) and then 25°C min^−1^ to 250°C and held for 1 min. Gas samples of 100 μl were withdrawn directly from the sampling flasks using a pressure lockable gastight glass syringe (SGE Analytical Science, Ringwood VIC, Australia) and injected into the GC. Compounds were quantified by comparison of the peak area of the unknown with an eight‐point calibration curve with a lower detection limit of 0.2 μM;. Calibration standards were made up in 60 ml serum vials.

For the analyses of acetate concentrations, water was filtered through a 0.2 μm syringe filter (Merck Millipore, Bayswater VIC, Australia) and subsequently 900 μl filtered water was acidified with 100 μl formic acid (10% v/v; Sigma‐Aldrich, Castle Hill NSW, Australia) to a pH < 2. Acetate (1 μl) was analysed by GC‐FID (Shimadzu, Rydalmere NSW, Australia) using a DB‐FFAP column (30 m × 0.32 mm; Agilent Technologies, Mulgrave, Australia) with helium (1 mL min^−1^) as carrier gas. Injection port was set at 250°C with split mode (1:30). Oven temperature was set to 60°C for 1 min and then 15°C min^−1^ to 250°C.

### Cell counts

Coal formation water samples were immediately fixed by the addition of glutaric dialdehyde (0.2 μm filtered, 2% final concentration) and stored at 4°C in the dark. Prior to analysis, an aliquot was diluted 1000‐fold in particle‐free phosphate‐buffered saline (0.9 g of NaCl, sodium phosphate buffer 15 mm, pH 7.4, 0.2 μm filtered), thoroughly shaken and transferred to a microscopic slide that was treated with a mounting medium (9.6% Mowiol 4‐88, Sigma‐Aldrich, Castle Hill NSW, Australia and 24% glycerol) prior to applications. Cells were stained using SybrGreen I (Sigma‐Aldrich, Castle Hill NSW, Australia), and counting was performed using a BX51 epifluorescence microscopy (Olympus, Notting Hill VIC, Australia) as described by Lunau *et al*. (2005).

### DNA extraction

After 17 and 25 months, DNA was extracted from 2 g of well sediment using the phenol/chloroform extraction method as described by Lueders and co‐workers (Lueders *et al*., 2004). Subsequently, DNA was precipitated using polyethylene glycol 6000 (Sigma‐Aldrich, St Louis MI, United States), and the DNA pellet was washed once with 70% ethanol and dissolved in 50 μl elution buffer (Qiagen, Venlo, the Netherlands). Three parallel extractions were carried out, and extracts were pooled for each incubation treatment. DNA concentration and purity were determined by standard agarose gel electrophoresis and fluorometrically using RiboGreen assays (Qubit Assay Kit, Invitrogen, Waltham MA, United States) according to the manufacturer's instructions.

### DNA sequencing and data processing

Both the 17 and 25 month DNA samples were used for 16S rRNA gene amplicon sequencing on the IonTorrent PGM™. Amplification of the V3‐V4 region of the bacterial 16S rRNA gene was performed using universal primers Bac F341 (5′‐CCTACGGGNGGCWGCAG‐3′) and Bac785R (5′‐GACTACHVGGGTATCTAATCC‐3′) (Klindworth *et al*., 2013) for 25 cycles. Archaeal 16S rRNA genes were amplified with the universal archaeal primers Archf349 (5′‐ GYGCASCAGKCGMGAAW‐3′) and Archr789 (5′‐GGACTACVSGGGTATCTAAT‐3′) (Klindworth *et al*., 2013) for 30 cycles. Both PCR amplifications were performed by 10 min 98°C initialization, 25/30 cycles of 1 min denaturation at 95°C, 1 min of annealing at 60°C, 2 min of elongation at 72°C and a 10 min final elongation step at 72°C. PCR products were purified using the QIAquick PCR Purification Kit (Qiagen, Venlo, the Netherlands) in two elution steps. 20 μl 55°C Milli‐Q was added to the spin column and incubated for 2 min prior to centrifugation. Next, the eluate was put onto the spin column, incubated at 55°C for 2 min and centrifuged again as described in the manual. A 10 cycle nested PCR with IonTorrent adapters was performed on the purified PCR products using the same PCR protocol. After PCR purification with the QIAquick PCR Purification Kit as described above, PCR products were used for library preparation and sequencing steps according to the manufacturer's instructions (Life Technologies, Carlsbad CA, United States). Amplicon sequences were quality checked for chimeras and clustered into OTUs with a 97% identity cut‐off value using the 454 SOP (http://www.mothur.org/) (Schloss *et al*., 2009) with IonTorrent modified protocols. Chimeras were checked with the Uchime algorithm (Edgar *et al*., 2011). Taxonomy was assigned against the silva nr v123 database using the MOTHUR taxonomy assigner (Schloss *et al*., 2009). Data visualization was performed using the ‘vegan’ package in r (Oksanen *et al*., 2017).

Metagenome sequencing of the 25‐month DNA sample was performed on the IonTorrent PGM™. All library preparation and sequencing steps were performed according to the manufacturer's instructions (Ion PGM™ Template OT2 400 Kit, Life Technologies, Carlsbad CA, United States). 100 ng genomic DNA was sheared in four‐one minute shear one minute cool down cycles using a Bioruptor^®^ Sonicator (Diagenode, Denville NJ, United States). The library was prepared using the Ion Plus Fragment Library Kit protocol. Size distribution and concentration of the library was determined using the Agilent 2100 Bioanalyzer (Agilent Technologies Inc., Santa Clara CA, United States). Sequencing was performed using the Ion 318 Chip Kit v2.

Raw reads were quality checked using the CLC Genomics Workbench 8.0 (CLCbio, Aarhus, Denmark). Ambiguous sequences were trimmed off using an ambiguous limit of 2 and a trim limit of 0.5. On both the 3′ and 5′ sides, the three terminal nucleotides were discarded. To reduce noise, reads were subsequently filtered, targeting reads with a length between 30 and 400 nt (5 153 485 reads) for *de novo* assembly and 100–400 nt (4 884 119 reads) for 16S rRNA and functional gene analysis. For *de novo* assembly, minimum contig size was set to 1000, word size to 35 and bubble size to 5000. Values for mismatch, insertion and deletion cost were set to 2, 3 and 3, respectively, resulting in a total of 12 527 contigs with an average length of 2624 base pairs. 85% of the reads (1.1 Gbp) were assigned to a specific contig based on a length and similarity fraction of 50% and 80% respectively.

### Analysis of environmental genomes

The assembled contig data set was analysed based on average read coverage and GC content per contig using r (https://www.r-project.org/) (R Core Team, 2014) and rstudio v3 (https://www.rstudio.com/) (RStudio Team, 2014) with packages ‘ggplot2’ (Wickham, 2009) and ‘rcolorbrewer 1.1‐2’ (Neuwirth, 2011) (https://cran.r-project.org/web/packages/). The *Geobacter* environmental draft genome bin was extracted based on a GC content between 0.525 and 0.65 and a sequencing depth between 100 and 325. The resulting bin contained 139 contigs of which 118 were most identical to *Geobacter metallireducens* GS‐15 (97% of bases, 3.4 Mbp, ≥ 99.9% nt identity). CheckM (Parks *et al*., 2015) confirmed presence of a single, 91.0% complete non‐heterogeneous strain. Identity of the draft genome contigs was determined by performing nucleotide blast searches against the nt/nr collection database. The methanogen environmental draft genome bin was extracted based on a GC content between 0.475 and 0.575 and a sequencing depth between 3 and 10. The resulting bin contained 459 contigs of which 353 contigs were most identical to *Methanothrix concilii* GP6 (86% of bases, 2.3 Mbp, ≥ 79.9% nt identity). CheckM showed strain heterogeneity of 11.1% and completeness of 76.8%, indicating the bin likely contains a core genome of *Methanothrix* species. Nucleotide blast on nine contigs with sequencing depth > 500 indicated best hits to *Geobacter metallireducens* GS‐15 (six sequences, ≥ 99% identity) and *Azoarcus* sp. (three sequences, ≥ 94% identity).

### Phylogenetic 16S and 18S rRNA gene analysis

For the general species composition analysis, quality trimmed reads were mapped against the Silva SSU non‐redundant database version 128 (https://www.arb-silva.de/) containing 645 151 reference sequences. Length and similarity fraction were set to 50% and 70%, respectively, and values for mismatch, insertion and deletion cost to 2, 3 and 3, resulting in a total of 28 774 mapped reads (0.59% of total). Sequences were submitted to the silvangs data analysis server 1.3.5 (Quast *et al*., 2012) and processed using default parameters. The species distribution pattern remains similar when additionally correcting for 16S rRNA gene copy numbers that are high in the Proteobacteria and Firmicutes phyla (Klappenbach *et al*., 2001; Acinas *et al*., 2004). Reads mapping to the SSU database were extracted per genus. *De novo* assembly was performed using automatic word and bubble size and stringent similarity and length fraction parameters of 0.95 to obtain near‐complete 16S sequences. Comparable approaches were taken previously (Bartram *et al*., 2011; Miller *et al*., 2011; Speth *et al*., 2016). Additionally, quality trimmed reads were mapped against the fungal internal transcribed spacer (ITS) RefSeq Targeted Loci database containing 5362 sequences (PRJNA177353) as described above (Alvarez *et al*., 2017).

### Functional analyses

Draft genome and metagenome annotations were performed using prokka version 1.10 with standard parameters (Seemann, 2014). The *Geobacter* environmental draft genome was first annotated against a *Geobacter* reference data set containing nine annotated genomes. Contig sequences were uploaded to the kegg Automated Annotation Server (KAAS) (Moriya *et al*., 2007) and analysed using 40 most related reference organisms. Specific protein sequence data sets for metal reduction, complex organic compounds degradation and genus specific genes were manually selected and downloaded from the uniprot database (http://www.uniprot.org/) (The UniProt Consortium, 2014). For the analysis of the fungal community, fungal peroxidase databases were made for lignin, manganese and versatile peroxidases by downloading nucleotide data from the NCBI database, filtering for fungal sequences ≤ 100 000 bp. Reference databases were imported in CLC and mapped against the genomic data sets using the clc blastx tool using the standard parameters: expect: 10.0, match: 2, mismatch: ‐3, gap costs existence: 5, gap cost extension: 2. Artemis 16.0.0 was used for visualization of the annotation results (Rutherford *et al*., 2000). Methanogen and methanotroph functional gene analyses were performed using McrA, MmoX and PmoA/AmoA protein sequence databases and a non‐redundant reference database (Claudia Lüke, Radboud University, personal communication). Analysis of trimmed reads was performed using the blastx (BLAST 2.2.25+) algorithm with *e*‐value 0.0001 extracting first hits as target sequence. Functional gene and non‐redundant BLAST results with a bitscore > 125 were merged. All reads had a blast score ratio of > 0.8 (Lüke *et al*., 2016). Only McrA analysis resulted in significant hits.

### Nucleotide sequence accession numbers

All sequencing data were submitted to the GenBank databases under the BioProject PRJNA321375. The assembled IonTorrent data set from this study was submitted as Sequence Read Archive study under accession number SRP074879 (run SRR5328925). The amplicon data were submitted as SAMN07344736 (17 month archaeal), SAMN07344812 (17 month bacterial), SAMN07344862 (25 month archaeal) and SAMN07344863 (25 month bacterial).

## Conflict of interest

None declared.

## Supporting information


**Table S1.** Groundwater characteristics of the coal seam well measured at the start of the experiment (0 months).
**Table S2.** Overview of SSU seed sequence and protein sequence analyses.
**Fig. S1.** Schematic of a coal seam gas well.
