# Supplementary material for: Nutrient and acetate amendment leads to acetoclastic methane production and microbial community change in a non‐producing Australian coal well
Source: Microb Biotechnol. 2017 Sep 19;11(4):626–38. doi: 10.1111/1751-7915.12853 (PMC6011947; doi:10.1111/1751-7915.12853)
Supplement: Supplementary file 1 — Table S1. Groundwater characteristics of the coal seam well measured at the start of the experiment (0 months). Table S2. Overview of SSU seed sequence and protein sequence analyses. Fig. S1. Schematic of a coal seam gas well. [file MBT2-11-626-s001.pdf]

Supplementary Material

Supplementary Table S1: Groundwater characteristics of the coal seam well measured at the start of the experiment (0 months). IDL: instrument detection limit, ND: not detected, TOC: total organic carbon, IC: inorganic carbon. The instrument detection limits were 0.02 mg/L for B, Si and Mn, 0.2 mg/L for Fe and P, 0.5 mg/L for Ca, K, Mg, Na and S, 2 mg/L for F, NO_2, NO_3 and Br, and 5 mg/L for Cl and PO_4.

**Component**	**Concentration**
Boron	3.70 µM
Bromide	<IDL
Calcium	0.89 mM
Chloride	0.13 mM
Fluoride	ND
Iron	1.25 mM
Magnesium	0.57 mM
Manganese	7.28 µM
Nitrate	<IDL
Nitrite	<IDL
Phosphate	<IDL
Potassium	0.44 mM
Sodium	0.66 mM
Silicium	0.21 mM
Sulfate	1.04 mM
Sulfur	1.08 mM
TOC	1.50 mg/L
IC	21.68 mg/L

Supplementary Table S2: Overview of SSU seed sequence and protein sequence analyses. **Bold underlined**: protein sequence (**Xxx** or Xxx**A**) detected in the metagenome, standard: protein sequence not detected (XxxA). SSU seed sequence identity shows identities based on BLASTN against the nucleotide collection nr/nt database. Sequence identity shows protein sequence identities based on BLASTX analyses with genus-specific UniProt databases on genus level. *DP4 and Hildenborough are names for the same *Desulfovibrio vulgaris* strain.

**SSU rRNA gene analyses**		**Functional analyses on protein level**			
**SILVAngs identity**	**Metagenomic SSU seed sequence identity (nucleotide %)**	**Main function**	**Full protein names**	**Short name**	**Protein sequence identity**
Basidiomycota	*Laetiporus sulphureus* (97%)	Organic compound metabolism	lignin, manganese and versatile peroxidases		≥55%
Geobacter	*Geobacter metallireducens* GS-15 (100%)	metal reduction	Macrolide export protein A<br>Periplasmic cytochrome c<br>PpcA homolog<br>Outer membrane cytochrome c	Mac**A**,<br>Ppc**A**BC**D**E<br>Gmet_2902<br>OmcBCEFST	100%<br>67-82%<br>100%<br>-
		Organic compound metabolism	Benzoylsuccinyl-CoA thiolase (BbsAB), 2-[hydroxyl(Phenyl)methyl]-succinyl-CoA dehydrogenase (BbsCD), Succinyl(R)-benzylsuccinate coenzyme A transferase (BbsEF), (R)-benzylsuccinyl-CoA dehydrogenase (BbsG), (E)-2-benzylidenesuccinyl-CoA	Bbs**ABCDEFGH**	100%

			hydratase (BbsH)<br>6-oxocyclohex-1-ene-1-carbonyl-CoA hydrolase (BamA), benzoyl-CoA reductase (putative) (BamBCDEFGH), glutaryl-CoA dehydrogenase (BamM), cyclohexa1,5-dienecarbonyl-CoA hydratase (BamR), 6-hydroxycyclohex-1-ene-1-carbonyl-CoA dehydrogenase (BamQ), benzoate-coenzyme A ligase (BamY)	Bam**ABCDEFGHMRQY**	100%
		VFA metabolism	Formate dehydrogenase<br>Acetate kinase<br>Propionyl-CoA carboxylase<br>Butyrate kinase (putative)	**Fdn**<br>Ack**A**<br>**Pcc**<br>**Buk**	100%<br>100%<br>100%<br>100%
		Motility & electron transfer	Flagellin (FliC), flagellar hook-associated protein 2 (FliD), flagellar protein (FliS)<br>Geopilin domain 1+2 (PilA-N & PilA-C)	Fli**CDS**<br>Pil**A**	100%<br>100%
Azoarcus	*Azoarcus evansii* KB740 (99%)	Organic compound metabolism	Benzylsuccinate synthase (BssABC), benzylsuccinate synthase activating enzyme (BssD), chaperone (BssE)<br>4-hydroxybenzoyl-CoA reductase (HcrAB)<br>3-hydroxybenzoate-CoA ligase (HbcL)<br>Benzoate-CoA ligase (BzdA)	Bss**ABCDE**<br>Hcr**AB**<br>Hbc**L**<br>Bzd**A**	≥93%<br>≥81%<br>≥81%<br>94%
Thauera	*Thauera aromatica* K172 (100%)	Organic compound metabolism	Benzylsuccinate synthase (BssABC), benzylsuccinate synthase activating enzyme (BssD)<br>4-hydroxybenzoyl-CoA reductase (HcrABC)<br>3-hydroxybenzoate-CoA/4-hydroxybenzoate-CoA ligase (Hcl)	Bss**A**B**CD**<br>Hcr**A**B**C**<br>**Hcl**	100%<br>≥93%<br>≥63%
Desulfovibrio	*Desulfovibrio vulgaris*	Hydrogenases	Periplasmic [NiFe] hydrogenase (HydABC)	Hyd**ABC**	≥94%
		Dissimilatory	Sulfite reductase (DsvAB)	Dsv**AB**	≥96%

	DP4/Hildenborough* (100%)	sulfate reduction			
Bacillus	*Bacillus subterraneus* Et6/3 16S (99%)	Organic compound metabolism	Benzene 1, 2-dioxygenase<br>Catechol-2,3-dioxygenase<br>Phenolic acid decarboxylase	**-**<br>Cat**E**<br>**-**	60-68%<br>48%<br>39%
Methanosaeta	*Methanothrix concilii* GP-6 (99%)	Methane and acetate metabolism	Methyl-coenzyme M reductase (McrABCD)<br>Acetyl-coenzyme A synthetase (Acs)	Mcr**ABCD**<br>**Acs**	≥99%<br>≥99%

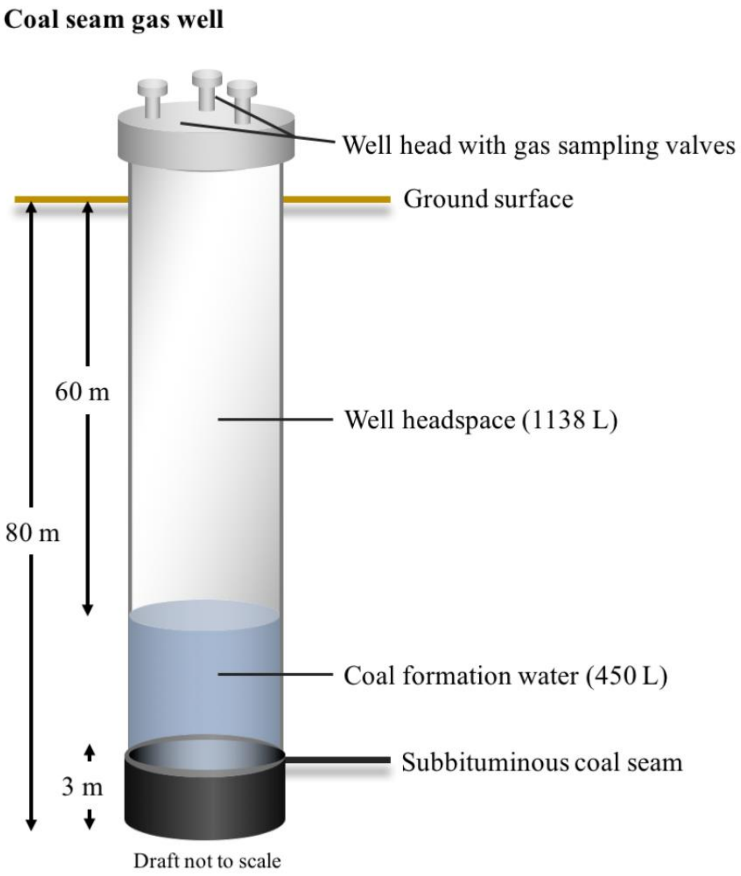

Supplementary Figure S1: Schematic of a coal seam gas well. Gas wells were drilled to 80 m depth, reaching 3 m into a subbituminous coal seam of the Sydney Basin, NSW, Australia. The well diameter was 158 mm and the total well volume was 1588 L (1138 L well headspace and 450 L coal formation water). The formation water submerged the coal and the lower 20 m of each well. The well head contained 3 gastight apertures for gas sampling. Gas samples were taken from the well head apertures and were transferred directly into 10 mL gastight serum vials using a gastight glass syringe.
